# Azithromycin Inhibits Mucus Hypersecretion from Airway Epithelial Cells

**DOI:** 10.1155/2012/265714

**Published:** 2012-04-23

**Authors:** Takeshi Shimizu, Shino Shimizu

**Affiliations:** Department of Otorhinolaryngology, Shiga University of Medical Science, Seta, Tsukinowa, Otsu, Shiga 520-2192, Japan

## Abstract

To examine the *in vivo* effects of the 15-member macrolide, azithromycin (AZM), on mucus hypersecretion, we induced hypertrophic and metaplastic changes of goblet cells in rat nasal epithelium by intranasal instillation of ovalbumin (OVA) in OVA-sensitized rats, or by intranasal lipopolysaccharides (LPS) instillation. Oral administration of AZM (5–10 mg/kg) or clarithromycin (CAM, 5–10 mg/kg) significantly inhibited OVA- and LPS-induced mucus production, whereas josamycin (JM) or ampicillin (ABPC) showed no effect. *In vitro* effects of AZM on airway epithelial cells were examined using NCI-H292 cells and human nasal epithelial cells cultured in air-liquid interface. Mucus secretion was evaluated by enzyme-linked immunosorbent assay using an anti-MUC5AC monoclonal antibody. AZM or CAM significantly inhibited tumor necrosis factor-*α* (TNF-*α*) (20 ng/mL)-induced MUC5AC secretion from NCI-H292 cells at 10^−6^–10^−7^ M, whereas JM or ABPC showed no effect. AZM significantly inhibited TNF-*α* (20 ng/mL)-induced MUC5AC secretion from human nasal epithelial cells at 10^−4^ M. MUC5AC mRNA expression was also significantly inhibited. These results indicate that the 15-member macrolide, AZM, exerts direct inhibitory effects on mucus secretion from airway epithelial cells and that it may be useful for the treatment of mucus hypersecretion caused by allergic inflammation and LPS stimulation.

## 1. Introduction

The 14-member macrolides, clarithromycin (CAM) and erythromycin (EM), and the 15-member macrolide, azithromycin (AZM), are widely used for the treatment of airway inflammation. Low-dose, long-term macrolide therapy has been reported to be very effective for patients with chronic airway diseases, such as diffuse panbronchiolitis [1], chronic bronchitis [2, 3], and chronic rhinosinusitis [4, 5]. It has been suggested that these effects depend on anti-inflammatory and immunomodulatory actions of 14- and 15-member macrolides rather than antibacterial one.

Hypersecretion of mucus is an important characteristic of these airway inflammations. The clinical effectiveness of macrolide therapy was represented by a significant reduction in the amount of secreted mucus. In our previous study, oral administration of CAM or EM significantly inhibited lipopolysaccharides- (LPS-) induced and antigen-induced mucus production in rat nasal epithelium, whereas 16-member macrolide, josamycin (JM), showed no effect. CAM and EM also inhibited mucus secretion from cultured airway epithelial cells, NCI-H292 cells, and human nasal epithelial cells cultured in air-liquid interface [6, 7]. These results indicate that the 14-member macrolides, CAM and EM, exert direct inhibitory effects on mucus secretion from airway epithelial cells. However, the inhibitory effect of 15-member macrolide, AZM, on mucus secretion is less well studied compared with CAM and EM.

 In the present study, to demonstrate the effects of AZM on mucus secretion from airway epithelial cells, we evaluated (1) the* in vivo *effects of AZM on antigen-induced and LPS-induced mucus production in rat nasal epithelium, and (2) the *in vitro* effects on tumor necrosis factor-*α*- (TNF-*α*-) induced mucus secretion from human mucoepidermoid carcinoma cells (NCI-H292 cells) and from human nasal epithelial cells cultured in air-liquid interface. Mucus secretion was evaluated by enzyme-linked immunosorbent assay (ELISA) using an anti-MUC5AC monoclonal antibody that recognizes peptide backbones of mucin. The effect on mRNA expression of MUC5AC gene was also examined.

## 2. Methods

### 2.1. Mucus Hypersecretion in Rat Nasal Epithelium

All experiments were approved by the Committee for the Care and Use of Laboratory Animals of Mie University School of Medicine. Sensitization and challenge of rats were performed as previously reported [8]. Male Fisher 344 rats (6 weeks old) were immunized with intraperitoneal injection of 200 *μ*g ovalbumin (OVA, grade V; Sigma Chemical Co., St. Louis, MO) and 10 mg of Al(OH)_3_ at days 1, 2, 3, and 11. At day 19, 0.1 mL saline containing 10 mg of OVA was instilled into nasal cavity for 3 days. For LPS stimulation, rats (9 weeks old) were intranasally instilled with 0.1 mL saline containing 0.1 mg LPS from *Escherichia coli *0111:B4 (Sigma) for 3 days [9].

AZM (5–10 mg/kg, Pfizer Pharmaceutical, Tokyo), CAM (5–10 mg/kg, Taisho Pharmaceutical, Tokyo), JM (10 mg/kg, Yamanouchi Pharmaceutical, Tokyo), or ampicillin (ABPC, 30 mg/kg, Sigma) in 0.5% carboxymethyl cellulose sodium salt was given orally 1 hour before the intranasal instillation of OVA or LPS for 3 days. Twenty-four hours after the last intranasal instillation of OVA or LPS, rats were sacrificed, and the nasal cavity was transversely sectioned at the level of incisive papilla. Paraffin sections were stained with alcian blue-periodic acid-Schiff and hematoxylin (AB-PAS-H).

### 2.2. Morphometry

The percentage area of AB-PAS-stained mucosubstance in the surface epithelium was determined with the image analyzer (SP 500, Olympus, Tokyo) [9]. The area of nasal epithelium was outlined, and the image analyzer determined the area of AB-PAS-stained mucosubstance within this reference area. The percentage area of mucosubstance per epithelial area was calculated over 2 mm (1 mm of each side of nasal septum ×2) of the basal lamina at the center of septal cartilage. Since the measured area of mucosubstance changes in the oblique section, the percent area of mucosubstance was used as a parameter of intraepithelial mucus production.

### 2.3. Cell Cultures

 A human mucoepidermoid carcinoma cell line, NCI-H292, was grown on plastic dish in RPMI 1640 medium containing 10% fetal bovine serum, penicillin streptomycin (50 U/mL-50 *μ*g/mL), and Hepes (25 mM).

Human nasal epithelial cells were obtained from nasal polyps from patients with chronic sinusitis. The dissociated epithelial cells were cultured in a serum-free hormone supplement medium according to a technique described previously [10]. An air-liquid interface was created when the cells became confluent, and the cultures were supplemented with medium containing 5 × 10^−8^ M retinoic acid.

 When the NCI-H292 cells become confluent, or at the 14-day culture in the air-liquid interface of nasal epithelial cells, tumor necrosis factor-*α* (TNF-*α*), and AZM, CAM, JM, or ABPC was added to the culture medium (pH7.2) for 24 hours, then the culture medium and total RNA were collected.

### 2.4. ELISA

 The culture medium were incubated at 40°C in a 96-well plate, until dry. Plates were blocked with 2% BSA for 1 hour, and then incubated with 50 *μ*L of mouse monoclonal MUC5AC antibody (1 : 100) for 1 hour. The wells were incubated with 100 *μ*L of horseradish peroxidase-goat anti-mouse IgG conjugate (1 : 10,000) for 1 hour. Color reaction was developed using 3,3′,5,5′-tetramethylbenzidine peroxidase solution. Absorbance was read at 450 nm.

### 2.5. Reverse Transcription-Polymerase Chain Reaction (RT-PCR)

 Total RNA was extracted from cultured cells, reverse transcribed, then the cDNA was amplified by PCR using the Superscript preamplification system kit (Gibco, Grand Island, NY). The MUC5AC cDNA was amplified using the sense primer 5′-CACCAAATACGCCAACAAGAC-3′ and the antisense primer 5′-CAGGGCCACGCAGCCAGAGAA-3′. The GAPDH cDNA was amplified using the sense primer 5′-CCACCCATGGCAAATTCCATGGCA-3′ and the antisense primer 5′-TCTAGACGGCAGGTCAGGTCCACC-3′.

### 2.6. Statistics

 All data are expressed as mean ± SD. The difference between variables was analyzed by the Mann-Whitney *U* test. Probability values of *P* < 0.05 were considered significant.

## 3. Results

### 3.1. *In Vivo* Effects on Mucus Production

 Intranasal instillation of OVA for 3 consecutive days induced hypertrophic and metaplastic changes of goblet cells in nasal septal epithelium of OVA-sensitized rats. Similar changes of goblet cells occurred after 3 days of LPS instillation. Only a few goblet cells were observed in control groups (untreated control, saline-instilled, and sham-sensitized rats challenged with saline or OVA, and OVA-sensitized rats challenged with saline).

**Figure 1:**
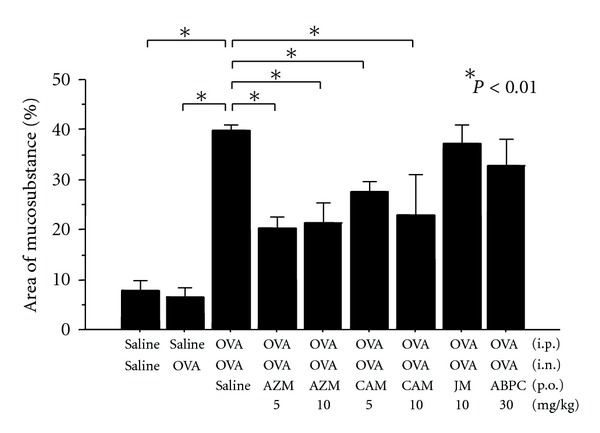
Effects of azithromycin (AZM, 5–10 mg/kg), clarithromycin (CAM, 5–10 mg/kg), josamycin (JM, 10 mg/kg), or ampicillin (ABPC, 30 mg/kg) on OVA-induced mucus production in OVA-sensitized rats (*n* = 6). Significant increase in intraepithelial mucosubstance occurred 24 hours after 3 days of OVA instillation. Oral administration of AZM or CAM significantly inhibited antigen-induced mucus production, whereas JM and ABPC had no effect.

**Figure 2:**
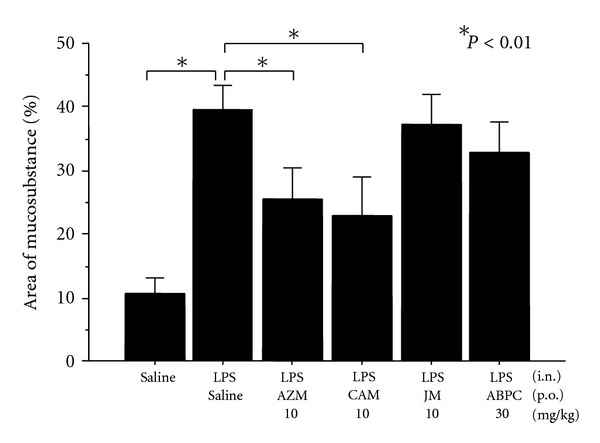
Effects of azithromycin (AZM, 10 mg/kg), clarithromycin (CAM, 10 mg/kg), josamycin (JM, 10 mg/kg), or ampicillin (ABPC, 30 mg/kg) on LPS-induced mucus production in rat nasal epithelium (*n* = 6). Significant increase in intraepithelial mucosubstance occurred 24 hours after 3 days of LPS instillation. Oral administration of AZM or CAM significantly inhibited LPS-induced mucus production, whereas JM and ABPC had no effect.

Oral administration of AZM (5–10 mg/kg) or CAM (5–10 mg/kg) significantly inhibited OVA-induced mucus production, whereas treatment with JM (16-member macrolide) or ABPC showed no significant effect (Figure 1). OVA-sensitized rats, challenged with OVA, showed significant infiltration of eosinophils in nasal septal mucosa, however, AZM had no effect on OVA-induced eosinophil infiltration. The number of eosinophils in nasal septal mucosa/8 mm (4 mm in each side ×2) was 2.6 ± 1.8 (saline control), 47.2 ± 17.7 (OVA-induced control), 51.4 ± 18.3 (AZM 5 mg/kg), and 44.4 ± 26,2 (AZM 10 mg/kg). LPS-induced mucus production was also significantly inhibited by the treatment with AZM (10 mg/kg) or CAM (10 mg/kg), whereas JM or ABPC showed no effect (Figure 2).

### 3.2. *In Vitro* Effects on Mucin Secretion

#### 3.2.1. NCI-H292 Cells

**Figure 3:**
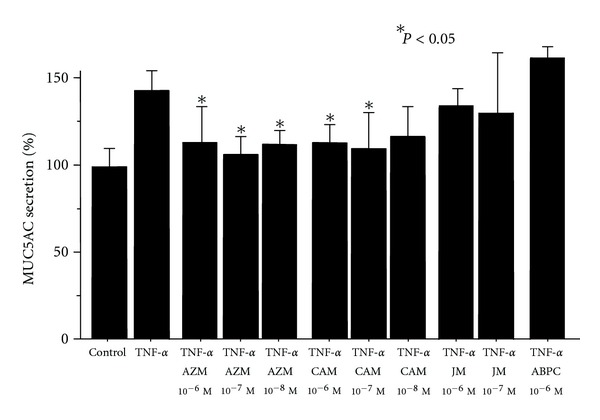
Effects of azithromycin (AZM), clarithromycin (CAM), josamycin (JM), and ampicillin (ABPC) on TNF-*α* (20 ng/mL)-induced MUC5AC secretion from NCI-H292 cells (*n* = 5). TNF-*α* stimulated mucin secretion. AZM and CAM significantly inhibited TNF-*α*-induced MUC5AC secretion, whereas JM and ABPC had no effect.

 TNF-*α* significantly stimulated mucin secretion from NCI-H292 cells. The percentage stimulation of MUC5AC secretion was 44.0% ± 8.6%. AZM showed an inhibitory effect on TNF-*α*-induced MUC5AC secretion at 10^−6^–10^−8 ^M. CAM (10^−6^–10^−7 ^M) also significantly inhibited TNF-*α*-induced mucin secretion, whereas JM (16-member macrolide) and ABPC showed no effects (Figure 3). 

#### 3.2.2. Human Nasal Epithelial Cells

**Figure 4:**
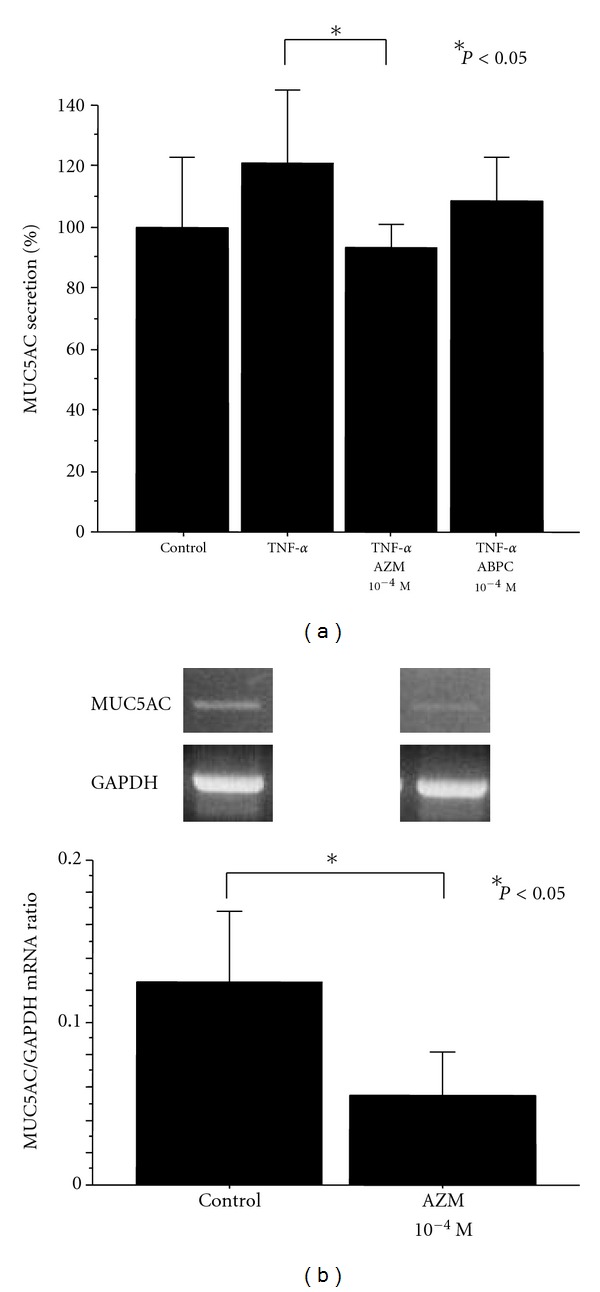
Effects of azithromycin (AZM) and ampicillin (ABPC) on TNF-*α* (20 ng/mL)-induced mucin secretion (a) and MUC5AC mRNA expression (b) from human nasal epithelial cells cultured at air-liquid interface (*n* = 5). (a) TNF-*α* stimulated MUC5AC secretion, and AZM significantly inhibited TNF-*α*-induced mucin secretion at 10^−4^ M, whereas ABPC showed no effect. (b) Total RNA was isolated and analyzed for MUC5AC and GAPDH mRNA expression by RT-PCR (*n* = 5). AZM significantly inhibited MUC5AC mRNA expression at 10^−4^ M as demonstrated by the MUC5AC/GAPDH ratio.

 At the 14-day culture in air-liquid interface condition, secretory cell differentiation was induced in about 25% of cultured cells [10]. The medium in the lower compartment did not react with MUC5AC. Only the samples collected from the apical side contained MUC5AC-reactive mucin, indicating that there was a polarity in mucin secretion. TNF-*α* (20 ng/mL) significantly stimulated MUC5AC secretion, and AZM significantly inhibited TNF-*α*-induced mucin secretion at 10^−4^ M from cultured human nasal epithelial cells, whereas ABPC showed no effect. Changes of MUC5AC gene expression were evaluated by RT-PCR, and AZM (10^−4 ^M) significantly inhibited MUC5AC mRNA expression of cultured human nasal epithelial cells (Figure 4).

## 4. Discussion

 In the present study, hypertrophic and metaplastic changes of goblet cells were induced in rat nasal epithelium by intranasal challenge with OVA in OVA-sensitized rats or by intranasal LPS instillation. A similar increase of epithelial mucosubstance occurred 24 hours after three days of OVA or LPS instillation. Oral administration of AZM (15-member macrolide) significantly inhibited antigen- or LPS-induced mucus production. These inhibitory effects are similar with CAM (14-member macrolide), whereas JM (16-member macrolide) or ABPC showed no effect. This is the first report showing the* in vivo *effects of AZM on mucus production in upper airways.

 Mucus hypersecretion associated with hypertrophy and metaplasia of epithelial secretory cells is a major characteristic of chronic airway diseases, and the clinical effectiveness of low-dose and long-term treatment with 14-member macrolides, CAM and EM, is represented by the significant reduction of the amount of secreted mucus, sputum, and rhinorrhea. Tamaoki and coworkers [11] have reported that erythromycin (EM) significantly inhibited mucus secretion in guinea pig trachea *in vivo*. In our previous studies [6, 7], CAM and EM inhibited antigen- and LPS-induced mucus production in rat nasal epithelium. CAM and EM showed the direct inhibitory effect on mucin secretion from cultured airway epithelial cells [6].

 The 15-member macrolide, AZM, also has an anti-inflammatory action, and AZM has been widely used for the treatment of patients with chronic airway inflammation, such as cystic fibrosis [12], chronic obstructive pulmonary disease [13], and bronchiolitis obliterans syndrome [14]. The meta-analysis study revealed that long-term use of AZM in cystic fibrosis patients improved the lung function, especially for *Pseudomonas aeruginosa*-colonized patients [12]. A large randomized placebo-controlled study revealed that long-term use of AZM decreased the risk of acute exacerbations of patients with chronic obstructive pulmonary diseases [13].

Several animal studies demonstrated that AZM attenuated many types of experimental airway inflammation caused by the allergic inflammation [15], by the inhalation of irritant gas, ozone [16], by the lung ischemia reperfusion injury [17], or by bacterial and viral infections [18, 19] in lower airways. In the present study, AZM also attenuated antigen- or LPS-induced mucus production in rat nasal epithelium. Many investigators demonstrated the anti-inflammatory action of AZM, which includes the immunomodulatory effects on inflammatory cells [19, 20], the modulation of cytokine production [21], and the inhibition of bacterial function and biofilm formation [22].

Recently, several *in vitro *studies have demonstrated the inhibitory effects of AZM on mucus secretion from airway epithelium. AZM inhibited MUC5AC expression and secretion from NCI-H292 cells, induced by human neutrophil peptide-1 and LPS [23], by *Pseudomonas aeruginosa*-derived N-(3-Oxododecanoyl) homoserine lactone [24], or by nontypable *Haemophilus influenza *and *Chlamydophilia pneumoniae* [25, 26]. AZM inhibited acetylcholine-induced MUC5AC release from swine airway submucosal gland cells [27]. In the present study, we examined the TNF-*α*-induced MUC5AC secretion from airway epithelial cells. TNF-*α* has been implicated in LPS-induced airway inflammation. LPS stimulation enhanced the TNF-*α*/*β* generation in rat lung [28], and TNF-*α* antagonist inhibited the LPS-induced mucus hypersecretion in rat nasal epithelium [29]. We found that AZM and CAM significantly inhibited TNF-*α*-induced MUC5AC secretion from NCI-H292 cells. AZM also inhibited mucin secretion from human nasal epithelial cells cultured in air-liquid interface, and MUC5AC mRNA expression was significantly inhibited. This is the first report showing the inhibitory effects of AZM on mucus secretion from normal human airway epithelial cells. These inhibitory actions appeared to be unique for 14- and 15-member macrolides because other antibiotics, JM (16-member macrolide) and ABPC, did not show any effect.

 In our previous study, the active concentrations of CAM and EM for the inhibition of mucin secretion are 10^−6^ to 10^−7^ M for NCI-H292 cells and 10^−4^ to 10^−5^ M for human nasal epithelial cells [6]. The different results may be caused by the different responses between mucoepidermoid carcinoma cells and normal nasal epithelial cells. In the present study, AZM showed the similar inhibitory effect on MUC5AC secretion from NCI-H292 cells and from human nasal epithelial cells. It is well known that the macrolide antibiotics achieve higher concentration in airway tissues, and the therapeutic concentrations are 10^−5^ to 10^−6^ M in tissues. In our *in vivo* study, oral administration of 5–10 mg/kg AZM or CAM significantly inhibited epithelial mucus production, and a previous study demonstrated that this is comparable with tissue concentration of 10^−5^ to 10^−6^ M in rats [30]. These results indicate that the *in vivo* effect of AZM or CAM is caused in some parts by the direct inhibitory effect on mucus secretion from the epithelial cells.

## 5. Conclusion

 We have induced hypertrophic and metaplastic changes of goblet cells in rat nasal epithelium by intranasal challenge with OVA in OVA-sensitized rat and by LPS instillation, and we have demonstrated in this model that AZM inhibits epithelial mucus production produced by allergic inflammation and by LPS stimulation. We have also demonstrated that AZM directly inhibits MUC5AC secretion from NCI-H292 cells and human nasal epithelial cells. These novel findings may explain the clinical efficacy of AZM in patients with chronic airway inflammation.
